# The Implications of HIV Treatment on the HIV-Malaria Coinfection Dynamics: A Modeling Perspective

**DOI:** 10.1155/2015/659651

**Published:** 2015-09-06

**Authors:** F. Nyabadza, B. T. Bekele, M. A. Rúa, D. M. Malonza, N. Chiduku, M. Kgosimore

**Affiliations:** ^1^Department of Mathematical Science, University of Stellenbosch, South Africa; ^2^South African Centre for Epidemiological Modelling and Analysis (SACEMA), Stellenbosch University, South Africa; ^3^Department of Biology, University of Mississippi, USA; ^4^Department of Mathematics, Kenyatta University, Kenya; ^5^Department of Applied Mathematics, National University of Science and Technology, Zimbabwe; ^6^Department of Basic Sciences, Botswana College of Agriculture, Botswana

## Abstract

Most hosts harbor multiple pathogens at the same time in disease epidemiology. Multiple pathogens have the potential for interaction resulting in negative impacts on host fitness or alterations in pathogen transmission dynamics. In this paper we develop a mathematical model describing the dynamics of HIV-malaria coinfection. Additionally, we extended our model to examine the role treatment (of malaria and HIV) plays in altering populations' dynamics. Our model consists of 13 interlinked equations which allow us to explore multiple aspects of HIV-malaria transmission and treatment. We perform qualitative analysis of the model that includes positivity and boundedness of solutions. Furthermore, we evaluate the reproductive numbers corresponding to the submodels and investigate the long term behavior of the submodels. We also consider the qualitative dynamics of the full model. Sensitivity analysis is done to determine the impact of some chosen parameters on the dynamics of malaria. Finally, numerical simulations illustrate the potential impact of the treatment scenarios and confirm our analytical results.

## 1. Introduction

The existence of parasites and pathogens has been documented in every free-living species [1–3]. Furthermore, most hosts are infected with multiple species of pathogenic organisms, leading to individual hosts which are coinfected [4, 5]. Coinfection occurs when two or more pathogen species exist within an individual at the same time [4]. Thus, the potential for interaction among cooccurring pathogens may result in major consequences for both the host and pathogen populations via negative impacts on host fitness or by altering pathogen transmission [5, 6].

Studies of coinfections with pathogens that impact humans have provided the most comprehensive exploration of the change of effects of coinfecting pathogens. Human Immunodeficiency Virus (HIV) and Acquired Immunodeficiency Syndrome (AIDS) represents one pathogen which has been documented to be particularly sensitive to coinfections. Specifically, increases in viral load as a result of recurrent or persistent coinfections, such as occuring when individuals are infected with both HIV/AIDS and malaria, tuberculosis, herpes simplex virus type 2, or helminths, may facilitate the increase in HIV transmission at both individual and population levels [7, 8].

The bidirectional and synergistical interactions of HIV and Malaria is particularly concerning because because of the geographic overlap between these two diseases in the Sub-Saharan region of Africa. In 2013, approximately 1.5 million (1.3 million–1.6 million) adults and children became infected with HIV [9]. Malaria epidemics are also widespread in this region where it is estimated that there were 198 million cases of malaria in 2013 and an estimated 584,000 deaths [10]. Furthermore, in the year 2010, an estimated 216 million clinical episodes of malaria and 650,000 deaths were registered in the world. Africa has the biggest disease burden, where an estimated 91% of deaths occur in this region [11]. Since both diseases are endemic to this region, and the time scale of infection for both diseases can be several years, the issue of coinfection plays a prominent role in understanding disease dynamics for this region [12]. Recent theoretical analyses examining the relationship between malaria and HIV in Sub-Saharan Africa indicate that malaria disproportionately contributes to the increase in incidence of HIV in the region [13]. In fact, without the amplification effect caused by malaria, HIV decreases to extinction [13]. Furthermore, empirical evidence demonstrates that individuals who live in areas with high rates of the malaria parasite* Plasmodium falciparum* have approximately twice the risk of being HIV positive compared with individuals who live in areas with low* P. falciparum* parasite rates [14]. Despite the importance of understanding coinfection dynamics of these two diseases, theoretical models exploring these relationships indicate a large amount of change of variation surrounding the possible outcome of HIV/AIDS-malaria coinfection dynamics for disease incidence.

Recent theoretical work examining HIV-malaria coinfections has neglected a key element of the epidemiology of these diseases, primarily the inclusion of treatment for one or both diseases [7]. The main objective of this study is thus to investigate the role of HIV treatment on the dynamics of malaria. In this study we formulate a theoretical model to explore population dynamics of HIV/AIDS-malaria coinfections. We decompose the model into submodels to gain insights into the transmission dynamics of each infection. Additionally we extended our model to examine the role treatment (for both malaria and HIV/AIDS) plays in altering populations dynamics. Our model consists of 13 interlinked equations which allow us to explore multiple aspects of transmission and treatment. We begin by first describing the full model; then we evaluate the individual models for positivity and boundedness of solutions, calculate *R*_0_, the basic reproduction number, and determine the stability of both the disease-free and endemic equilibriums. Finally, we use simulations to explore population-level disease dynamics.

## 2. HIV-Malaria Coinfection Model

We considered a sexually active human population subdivided into the following subpopulations: being susceptible to all pathogens (*S*), being exposed to malaria only (*E*_*M*_), being infected with malaria only (*I*_*M*_), being infected with HIV only (*I*_*H*_), having developed AIDS (*A*_*H*_), being asymptomatic for AIDS but infected with malaria (*I*_*HM*_), and being symptomatic to both AIDS and malaria (*A*_*HM*_). In addition, we have different states to represent the different stages of HIV treatment: *T*_*H*_ and *T*_*A*_ represent proportion of individuals receiving HIV treatment from *I*_*H*_ and *A*_*H*_, respectively, and *T*_*HM*_ and *T*_*AM*_ are the coinfection states of the previous states mentioned.

We assume that individuals in state *A*_*HM*_ will first receive treatment for malaria before starting HIV treatment to minimize drug complication issues. We also assume the average recovery rate from malaria is less than a week, which is fast compared to the slow HIV dynamics, but consistent with observed recovery values for malaria. Newly sexually active individuals are recruited to the human population at a rate Λ_*H*_. Individuals in state *E*_*M*_ either progress to symptomatic stage at a rate *p* or recover naturally at a rate *ϕ*. The rate at which malaria infected individuals recover from malaria is *γ*. Here we introduce scaling parameters *ϵ*_1_ and *ϵ*_2_ for the recovery rates from states *I*_*HM*_ and *A*_*HM*_, respectively. These parameters are assumed to be less than 1, with *ϵ*_1_ > *ϵ*_2_ to account for the fact that coinfected individuals have compromised immune systems, whereas *η*_1_, *η*_2_, *η*_3_, and *η*_4_ (each assumed to be greater than 1) scale the force of infection term for malaria (*λ*_*M*_) to account for individuals' history of HIV/AIDS infection. The rates at which HIV infected individuals and those coinfected with malaria progress to AIDS stages (*A*_*H*_ and *A*_*HM*_, resp.) are represented by *α*_1_ and *α*_2_.

Since our main interest is to study the impact of HIV treatment on the reduction of malaria cases, we consider a hypothetical situation where individuals are adherent to HIV treatment. For this study, we assume that individuals who start treatment stay in the treatment program for the rest of their lives. Individuals die of malaria at a rate *δ*_*M*_, where *τ* > 1 indicates an increase in the risk of death due to coinfection with HIV for individuals in states *I*_*HM*_, *A*_*HM*_, *T*_*HM*_, and *T*_*AM*_. Similarly, we have a scaling parameter, *φ* < 1, for HIV/AIDS induced death due to treatment for *T*_*H*_ and *T*_*A*_, where *δ*_*H*_ is an HIV/AIDS induced death rate for individuals in *I*_*H*_. Individuals on HIV treatment coinfected with malaria can recover from malaria at a rate *q*.

The mosquito population *N*_*V*_ is subdivided into susceptible mosquitoes (*S*_*V*_), mosquitoes exposed to the parasite (*E*_*V*_), and infectious mosquitoes (*I*_*V*_). Susceptible mosquitoes are generated at a constant rate Λ_*V*_ and acquire malaria infection (following effective contacts with humans infected with malaria) at force of infection rate *λ*_*V*_. Mosquitoes are assumed to suffer natural death at a rate *μ*_*V*_, regardless of their infection status. Newly infected mosquitoes are moved into the exposed class (*E*_*V*_) and progress to the class of symptomatic mosquitoes (*I*_*V*_) following the development of symptoms at a rate *θ*_*V*_.

**Figure 1:**
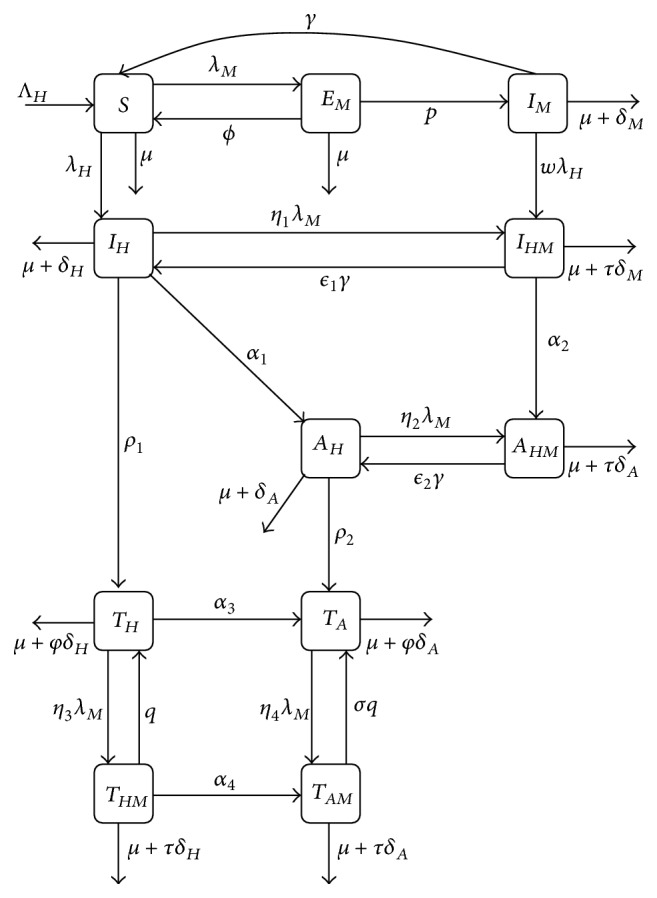
HIV-malaria coinfection model (the human population).

**Figure 2:**
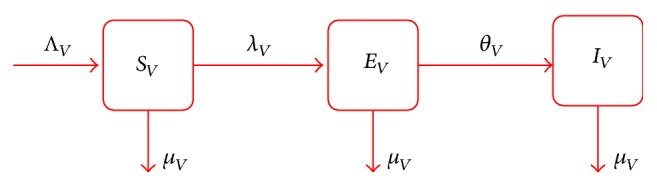
Vector population.

Here we construct a system of equations to model the disease dynamics of HIV/AIDS and malaria. The assumptions given above and the schematic diagrams (Figures 1 and 2) result in the following system:

(1) $$\begin{array}{c} \frac{dS}{dt}=\Lambda_{H}+\phi E_{M}+\gamma I_{M}-\left(\lambda_{M}+\lambda_{H}+\mu \right)S,\\ \frac{dE_{M}}{dt}=\lambda_{M}S-\left(\phi +p+\mu \right)E_{M},\\ \frac{dI_{M}}{dt}=pE_{M}-\left(\gamma +w\lambda_{H}+\mu +\delta_{M}\right)I_{M},\\ \frac{dI_{H}}{dt}=\lambda_{H}S+\epsilon_{1}\gamma I_{HM}-\left(\eta_{1}\lambda_{M}+\rho_{1}+\alpha_{1}+\mu +\delta_{H}\right)I_{H},\\ \frac{dT_{H}}{dt}=\rho_{1}I_{H}+qT_{HM}-\left(\alpha_{3}+\eta_{3}\lambda_{M}+\mu +\varphi \delta_{H}\right)T_{H},\\ \frac{dA_{H}}{dt}=\alpha_{1}I_{H}+\epsilon_{2}\gamma A_{HM}-\left(\eta_{2}\lambda_{M}+\rho_{2}+\mu +\delta_{A}\right)A_{H},\\ \frac{dT_{A}}{dt}=\rho_{2}A_{H}+\alpha_{3}T_{H}+\sigma qT_{AM}-\left(\eta_{4}\lambda_{M}+\mu +\varphi \delta_{A}\right)T_{A},\\ \frac{dI_{HM}}{dt}=\eta_{1}\lambda_{M}I_{H}+w\lambda_{H}I_{M}-\left(\epsilon_{1}\gamma +\alpha_{2}+\mu +\tau \delta_{M}\right)I_{HM},\\ \frac{dA_{HM}}{dt}=\alpha_{2}I_{HM}+\eta_{2}\lambda_{M}A_{H}-\left(\epsilon_{2}\gamma +\mu +\tau \delta_{A}\right)A_{HM},\\ \frac{dT_{HM}}{dt}=\eta_{3}\lambda_{M}T_{H}-\left(q+\alpha_{4}+\mu +\tau \delta_{H}\right)T_{HM},\\ \frac{dT_{AM}}{dt}=\eta_{4}\lambda_{M}T_{A}+\alpha_{4}T_{HM}-\left(\sigma q+\mu +\tau \delta_{A}\right)T_{AM},\\ \frac{dS_{V}}{dt}=\Lambda_{V}-\left(\lambda_{V}+\mu_{V}\right)S_{V},\\ \frac{dE_{V}}{dt}=\lambda_{V}S_{V}-\left(\theta_{V}+\mu_{V}\right)E_{V},\\ \frac{dI_{V}}{dt}=\theta_{V}E_{V}-\mu_{V}I_{V}. \end{array}$$

The total human and vector populations are, respectively, given by

(2) $$\begin{array}{c} N_{H}=S+E_{M}+I_{M}+I_{H}+I_{HM}+A_{H}+A_{HM}+T_{H}+T_{A}+T_{HM}+T_{AM}, \end{array}$$

(3) $$\begin{array}{c} N_{V}=S_{V}+E_{V}+I_{V}. \end{array}$$

Susceptible individuals get infected with HIV at a rate *λ*_*H*_ and with malaria at a rate *λ*_*M*_, where

(4) $$\begin{array}{c} \lambda_{H}=\beta_{H}\frac{I_{H}+\theta_{1}I_{HM}+\theta_{2}A_{H}+\theta_{3}A_{HM}+\theta_{4}T_{H}+\theta_{5}T_{A}+\theta_{6}T_{HM}+\theta_{7}T_{AM}}{N_{H}},\\ \lambda_{M}=\beta_{M}b_{M}\frac{I_{V}}{N_{H}}. \end{array}$$

Mosquitoes acquire malaria parasite from malaria infected individuals at a rate *λ*_*V*_ following effective transmission probability *β*_*V*_ and mosquito biting rate *b*_*M*_ given by the expression

(5) $$\begin{array}{c} \lambda_{V}=\beta_{V}b_{M}\frac{I_{M}+q_{1}I_{HM}+q_{2}A_{HM}+q_{3}T_{HM}+q_{4}T_{AM}}{N_{H}}, \end{array}$$

where *θ*'s and *q*'s are factors indicating the differential contribution for the HIV and malaria infection, respectively. *β*_*H*_ is the effective contact rate for HIV infection, *β*_*V*_ is the transmission probability for mosquito infection, and *b*_*M*_ is the biting rate of mosquitoes. Summary of parameters in the HIV-malaria coinfection model is shown in Notations section.

Before analyzing the dynamics of the full model, we first consider the submodels (HIV-only and malaria-only) separately.

## 3. HIV-Only Model

The HIV-only model is obtained by setting states of malaria infection and coinfection equal to zero in (1) and is described by the following equations:

(6) $$\begin{array}{c} \frac{dS}{dt}=\Lambda_{H}-\left(\lambda_{H}+\mu \right)S,\\ \frac{dI_{H}}{dt}=\lambda_{H}S-\left(\rho_{1}+\alpha_{1}+\mu +\delta_{H}\right)I_{H},\\ \frac{dA_{H}}{dt}=\alpha_{1}I_{H}-\left(\rho_{2}+\mu +\delta_{A}\right)A_{H},\\ \frac{dT_{H}}{dt}=\rho_{1}I_{H}-\left(\alpha_{3}+\mu +\varphi \delta_{H}\right)T_{H},\\ \frac{dT_{A}}{dt}=\alpha_{3}T_{H}+\rho_{2}A_{H}-\left(\mu +\varphi \delta_{A}\right)T_{A}, \end{array}$$

where *λ*_*H*_ is the force of infection term given by

(7) $$\begin{array}{c} \lambda_{H}=\beta_{H}\frac{I_{H}+\theta_{2}A_{H}+\theta_{4}T_{H}+\theta_{5}T_{A}}{N_{H}},\;\text{with }N_{H}=S+I_{H}+A_{H}+T_{H}+T_{A}. \end{array}$$

*Model Properties.* Following [15], we have the following theorem on the positivity and invariance of system (6).

**Theorem 1 .**

(1) The region *ℛ*_*ℋ*_ given by

(8) $$\begin{array}{c} R_{H}=\left\{S\left(t\right),I_{H}\left(t\right),A_{H}\left(t\right),T_{H}\left(t\right),T_{A}\left(t\right)\in R_{+}^{5}:N_{H}\leq \frac{\Lambda_{H}}{\mu }\right\} \end{array}$$

is positively invariant and attracting with respect to model system (6).

(2) The solutions (*S*(*t*), *A*_*H*_(*t*), *T*_*H*_(*t*), *T*_*A*_(*t*)) of model (6) are positive for all *t* ≥ 0 for any given nonnegative initial conditions.

### 3.1. The Basic Reproduction Number, *R*_0*H*_

We use the next-generation matrix method to determine the basic reproduction number, *R*_0*H*_, of the submodel.

The basic reproduction number is given by

(9) $$\begin{array}{c} R_{0H}=\zeta \left(FV^{-1}\right)=\frac{\beta_{H}}{K}\left(1+\frac{\theta_{2}\alpha_{1}}{L}+\frac{\theta_{4}\rho_{1}}{M}+\frac{\theta_{5}\alpha_{3}\rho_{1}}{MN}+\frac{\theta_{5}\alpha_{1}\rho_{2}}{LN}\right), \end{array}$$

where *K* = *δ*_*H*_ + *μ* + *α*_1_ + *ρ*_1_, *L* = *μ* + *δ*_*A*_ + *ρ*_2_, *M* = *μ* + *φδ*_*H*_ + *α*_3_, *N* = *μ* + *φδ*_*A*_.

### 3.2. Stability of the Disease-Free Equilibrium

The disease-free equilibrium point, *ε*_*H*0_ of the HIV-only model, is obtained by setting all the infectious classes to zero so that

(10) $$\begin{array}{c} \varepsilon_{H0}=\left(S,I_{H},A_{H},T_{H},T_{A}\right)=\left(\frac{\Lambda_{H}}{\mu },0,0,0,0\right). \end{array}$$

Local stability of *ε*_*H*0_ is guaranteed by using Theorem  2 in [16]. However, global stability can be established using the method first outlined in [17]. We thus have the following theorem.

**Theorem 2 .**

The fixed point *U*_0_ = (*X* ^*∗*^; 0) = (Λ_*H*_/*μ*, 0,0, 0,0) is a globally asymptotic stable equilibrium of system (6) if *R*_0*H*_ < 1.

### 3.3. The Endemic Equilibrium

The endemic equilibrium point of the system is given by

(11) $$\begin{array}{c} \varepsilon_{H}^{*}=\left(S^{*},I_{H}^{*},A_{H}^{*},T_{H}^{*},T_{A}^{*}\right), \end{array}$$

where

(12) $$\begin{array}{c} S^{*}=\frac{\Lambda_{H}}{\mu }+\frac{K\Lambda_{H}\left(R_{0H}-1\right)}{\mu \left(\beta_{H}\Gamma -\mu \psi \right)},\\ I_{H}^{*}=\frac{\Lambda_{H}\left(R_{0H}-1\right)}{\beta_{H}\Gamma -\mu \psi },\\ A_{H}^{*}=\frac{\alpha_{1}\Lambda_{H}\left(R_{0H}-1\right)}{L\left(\beta_{H}\Gamma -\mu \psi \right)},\\ T_{H}^{*}=\frac{\rho_{1}\Lambda_{H}\left(R_{0H}-1\right)}{M\left(\beta_{H}\Gamma -\mu \psi \right)},\\ T_{A}^{*}=\frac{\Lambda_{H}\left(L\alpha_{3}\rho_{1}+M\alpha_{1}\rho_{2}\right)\left(R_{0H}-1\right)}{LMN\left(\beta_{H}\Gamma -\mu \psi \right)}, \end{array}$$

with

(13) $$\begin{array}{c} \Gamma =1+\frac{\theta_{2}\alpha_{1}}{L}+\frac{\theta_{4}\rho_{1}}{M}+\frac{\theta_{5}\alpha_{3}\rho_{1}}{MN}+\frac{\theta_{5}\alpha_{1}\rho_{2}}{LN},\\ \psi =\frac{K}{\mu }-\left(1+\frac{\alpha_{1}}{L}+\frac{\rho_{1}}{M}+\frac{\alpha_{3}\rho_{1}}{MN}+\frac{\alpha_{1}\rho_{2}}{LN}\right). \end{array}$$

**Remark 3 .**

Given that *R*_0*H*_ = *β*_*H*_Γ/*K* is the basic reproduction number, when *R*_0*H*_ = 1, *ε*_*H*_^*∗*^ reduces to *ε*_*H*0_.

We use the Center Manifold Theory described in [18] to study the local stability of the endemic equilibrium point (close to *R*_0*H*_ = 1). We apply Theorem  4.1 duplicated as Theorem A.1 in the Appendix  for ease of reference. Model system (6) can be rewritten as follows using change of variables:

(14) $$\begin{array}{c} \frac{dX}{dt}=F=\left(f_{1},f_{2},f_{3},f_{4},f_{5}\right), \end{array}$$

such that

(15) $$\begin{array}{c} x_{1}^{\prime }\left(t\right)=f_{1}=\Lambda_{H}-\left(\lambda_{H}+\mu \right)x_{1},\\ x_{2}^{\prime }\left(t\right)=f_{2}=\lambda_{H}x_{1}-\left(\rho_{1}+\alpha_{1}+\mu +\delta_{H}\right)x_{2},\\ x_{3}^{\prime }\left(t\right)=f_{3}=\alpha_{1}x_{2}-\left(\rho_{2}+\mu +\delta_{A}\right)x_{3},\\ x_{4}^{\prime }\left(t\right)=f_{4}=\rho_{1}x_{2}-\left(\alpha_{3}+\mu +\varphi \delta_{H}\right)x_{4},\\ x_{5}^{\prime }\left(t\right)=f_{5}=\alpha_{3}x_{4}+\rho_{2}x_{3}-\left(\mu +\varphi \delta_{A}\right)x_{5}. \end{array}$$

We choose *β*_*H*_ as a bifurcation parameter. It can be shown that the Jacobian matrix at *β*_*H*_ = *β*_*H*_^*∗*^ has a right eigenvector associated with the zero eigenvalue. Evaluation of the right and left eigenvectors aids the determination of the coefficients** a** and** b**. We thus have

(16) $$\begin{array}{c} a=\overset{n}{\underset{k,i,j=1}{\sum }}v_{k}w_{i}w_{j}\frac{\partial^{2}f_{k}}{\partial x_{i}\partial x_{j}}\left(0,0\right), \end{array}$$

which gives

(17) $$\begin{array}{c} a=-\frac{1}{\Lambda_{H}}\beta_{H}\mu^{3}{\left(\mu +\varphi \delta_{A}\right)}^{2}\left(\mu +\alpha_{3}+\varphi \delta_{H}\right)\left(\mu +\delta_{A}+\rho_{2}\right)\left(2\left(\mu +\varphi \delta_{A}\right){\left(\mu +\alpha_{3}+\varphi \delta_{H}\right)}^{2}+\left(\mu +\alpha_{3}+\varphi \delta_{H}\right)\left(\left(\mu +\varphi \delta_{A}\right)\left(1+\theta_{4}\right)+\alpha_{3}\left(1+\theta_{5}\right)\right)\rho_{1}+\left(\left(\alpha_{3}+2\left(\mu +\varphi \delta_{A}\right)\right)\theta_{4}+\alpha_{3}\theta_{5}\right)\rho_{2}\right){\left(\mu +\delta_{A}+\rho_{2}\right)}^{2}+\alpha_{1}^{2}{\left(\mu +\alpha_{3}+\varphi \delta_{H}\right)}^{2}\left(2\left(\mu +\varphi \delta_{A}\right)\theta_{2}+\left(\theta_{2}+\theta_{5}\right)\rho_{2}\right)+\alpha_{1}\left(\mu +\alpha_{3}+\varphi \delta_{H}\right)\left(\mu +\delta_{A}+\rho_{2}\right)\left(\left(\left(\mu +\varphi \delta_{A}\right)\left(\left(\mu +\varphi \delta_{H}\right)\left(1+\theta_{2}\right)+\left(\theta_{2}+\theta_{4}\right)\rho_{1}\right)+\left(\left(\mu +\varphi \delta_{H}\right)\left(1+\theta_{5}\right)+\left(\theta_{4}+\theta_{5}\right)\rho_{1}\right)\rho_{2}+\alpha_{3}\left(\mu +\varphi \delta_{A}\left(1+\theta_{2}\right)+\theta_{2}\left(\mu +\rho_{1}\right)+\rho_{2}+\theta_{5}\left(\rho_{1}+\rho_{2}\right)\right)\right)\right). \end{array}$$

Similarly,

(18) $$\begin{array}{c} b=\overset{n}{\underset{k,i=1}{\sum }}v_{k}w_{i}\frac{\partial^{2}f_{k}}{\partial x_{i}\partial \beta_{H}}\left(0,0\right) \end{array}$$

results in

(19) $$\begin{array}{c} b=\mu \left(\mu +\varphi \delta_{A}\right)\left(\mu +\alpha_{3}+\varphi \delta_{H}\right)\left(\mu +\delta_{A}+\rho_{2}\right)\left(\alpha_{1}\left(\mu +\varphi \delta_{A}\right)\left(\mu +\alpha_{3}+\varphi \delta_{H}\right)\theta_{2}+\left(\mu +\varphi \delta_{A}\right)\left(\mu +\alpha_{3}+\varphi \delta_{H}\right)\left(\mu +\delta_{A}+\rho_{2}\right)+\left(\mu +\varphi \delta_{A}\right)\theta_{4}\rho_{1}\left(\mu +\delta_{A}+\rho_{2}\right)+\theta_{5}\left(\alpha_{1}\left(\mu +\alpha_{3}+\varphi \delta_{H}\right)\rho_{2}+\alpha_{3}\rho_{1}\left(\mu +\delta_{A}+\rho_{2}\right)\right)\right). \end{array}$$

Thus, *a* < 0 and *b* > 0. We thus have the following theorem.

**Theorem 4 .**

The endemic equilibrium is locally asymptotically stable for *R*_0*H*_ near 1.

## 4. Malaria-Only Model

In a similar way, if we set states of HIV and coinfection equal to zero in (1), we obtain malaria-only model described by the following system of equations:

(20) $$\begin{array}{c} \frac{dS}{dt}=\Lambda_{H}+\phi E_{M}+\gamma I_{M}-\left(\lambda_{M}+\mu \right)S,\\ \frac{dE_{M}}{dt}=\lambda_{M}S-\left(\phi +p+\mu \right)E_{M},\\ \frac{dI_{M}}{dt}=pE_{M}-\left(\gamma +\mu +\delta_{M}\right)I_{M},\\ \frac{dS_{V}}{dt}=\Lambda_{V}-\left(\lambda_{V}+\mu_{V}\right)S_{V},\\ \frac{dE_{V}}{dt}=\lambda_{V}S_{V}-\left(\theta_{V}+\mu_{V}\right)E_{V},\\ \frac{dI_{V}}{dt}=\theta_{V}E_{V}-\mu_{V}I_{V}. \end{array}$$

The force of infection terms for malaria infection from mosquito to human and vice versa is given by *λ*_*M*_ and *λ*_*V*_, respectively, where

(21) $$\begin{array}{c} \lambda_{M}=\beta_{M}b_{M}\frac{I_{V}}{N_{H}},\\ \lambda_{V}=\beta_{V}b_{M}\frac{I_{M}}{N_{H}}. \end{array}$$

### 4.1. Model Properties

The following results can easily be established on the positivity and boundedness of the solutions of system (20) in the feasible region

(22) $$\begin{array}{c} \Omega \subseteq \Omega_{H}\times \Omega_{V}, \end{array}$$

where

(23) $$\begin{array}{c} \Omega_{H}=\left\{\left(S,E_{M},I_{M}\right)\;|\;N_{H}\leq \frac{\Lambda_{H}}{\mu },\;S\leq \frac{\Lambda_{H}^{2}\mu_{V}\left(\phi +\gamma +\mu \right)}{\mu \left(\beta_{M}b_{M}\Lambda_{V}\mu +\left(\phi +\gamma +\mu \right)\Lambda_{H}\mu_{V}\right)},\;E_{M}\leq \frac{\beta_{M}b_{M}\Lambda_{V}\left(\phi +p+\mu \right)}{\left(\phi +p+\mu \right)\left(\beta_{M}b_{M}\Lambda_{V}\mu +\left(\phi +\gamma +\mu \right)\Lambda_{H}\mu_{V}\right)},\;I_{M}\geq 0\right\},\\ \Omega_{V}=\left\{\left(S_{V},E_{V},I_{V}\right)\;|\;N_{V}\leq \frac{\Lambda_{V}}{\mu_{V}},\;S_{V}\leq \frac{\Lambda_{V}}{\beta_{V}b_{M}+\mu_{V}},\;E_{V}\leq \frac{\beta_{V}b_{M}\Lambda_{V}}{\left(\beta_{V}b_{M}+\mu_{V}\right)\left(\theta_{V}+\mu_{V}\right)},\;I_{V}\geq 0\right\}. \end{array}$$

**Theorem 5 .**

(1) The solutions (*S*(*t*), *E*_*M*_(*t*), *I*_*M*_(*t*), *S*_*V*_(*t*), *E*_*V*_(*t*), *I*_*V*_(*t*)) of model (20) are positive for all *t* ≥ 0 for nonnegative initial conditions.

(2) All solutions (*S*(*t*), *E*_*M*_(*t*), *I*_*M*_(*t*), *S*_*V*_(*t*), *E*_*V*_(*t*), *I*_*V*_(*t*)) of model (20) are bounded.

### 4.2. The Basic Reproduction Number, *R*_0*M*_

The associated basic reproduction number *R*_0*M*_ of the model is given by

(24) $$\begin{array}{c} R_{0M}=\zeta \left(FV^{-1}\right)=\sqrt{R_{0M}^{h}R_{0M}^{v}}, \end{array}$$

where

(25) $$\begin{array}{c} R_{0M}^{h}=\frac{p\beta_{M}\mu }{\Lambda_{H}\left(\phi +p+\mu \right)\left(\gamma +\mu +\delta_{M}\right)},\\ R_{0M}^{v}=\frac{b_{M}^{2}\beta_{V}\theta_{V}\Lambda_{V}}{\mu_{V}^{2}\left(\theta_{V}+\mu_{V}\right)}. \end{array}$$

Here, *R*_0*M*_^*h*^ represents the contribution from the human population, while *R*_0*M*_^*v*^ represents the contribution from the vector population.

### 4.3. Stability of the Disease-Free Equilibrium

We begin by determining the disease-free equilibrium point *ε*_*M*0_ of the malaria-only model, by setting all the equations in (20) equal to zero so that

(26) $$\begin{array}{c} \varepsilon_{M0}=\left(S,E_{M},I_{M},S_{V},E_{V},I_{V}\right)=\left(\frac{\Lambda_{H}}{\mu },0,0,\frac{\Lambda_{V}}{\mu_{V}},0,0\right). \end{array}$$

Following Theorem  2 stated in [16], we present local stability property for the disease-free equilibrium in the following theorem.

**Theorem 6 .**

The disease-free equilibrium of system (20) is locally asymptotically stable whenever *R*_0*M*_ < 1 and unstable otherwise.

The global stability of (20) follows [17]. We thus have the following theorem.

**Theorem 7 .**

The fixed point $\bar{U_{0}}=(X^{*};0)=(\Lambda_{H}/\mu ,\Lambda_{V}/\mu_{V},0,0,0,0)$ is a globally asymptotic stable (g.a.s) equilibrium of system (20) if *R*_0*M*_ < 1.

### 4.4. The Endemic Equilibrium

The endemic equilibrium point of (20) is given by

(27) $$\begin{array}{c} \varepsilon_{M1}=\left(S^{*},E_{M}^{*},I_{M}^{*},S_{V}^{*},E_{V}^{*},I_{V}^{*}\right), \end{array}$$

where

(28) $$\begin{array}{c} S^{*}=\frac{\left(\gamma +\mu +\delta_{M}\right)\left(\phi +p+\mu \right)\Lambda_{H}}{\mu \left(\phi +p+\mu \right)\gamma +\mu +\delta_{M}+\lambda_{M}^{*}\left(\mu \left(p+\gamma +\mu \right)+\left(p+\mu \right)\delta_{M}\right)},\\ E_{M}^{*}=\frac{\lambda_{M}^{*}\Lambda_{H}\left(\gamma +\mu +\delta_{M}\right)}{\mu \left(\phi +p+\mu \right)\left(\gamma +\mu +\delta_{M}\right)+\lambda_{M}^{*}\left(\mu \left(p+\gamma +\mu \right)+\left(p+\mu \right)\delta_{M}\right)},\\ I_{M}^{*}=\frac{p\lambda_{M}^{*}\Lambda_{H}}{\mu \left(\phi +p+\mu \right)\left(\gamma +\mu +\delta_{M}\right)+\lambda_{M}^{*}\left(\mu \left(p+\gamma +\mu \right)+\left(p+\mu \right)\delta_{M}\right)},\\ S_{V}^{*}=\frac{\Lambda_{V}}{\lambda_{V}^{*}+\mu_{V}},\\ E_{V}^{*}=\frac{\lambda_{V}^{*}\Lambda_{V}}{\left(\lambda_{V}^{*}+\mu_{V}\right)\left(\theta_{V}+\mu_{V}\right)},\\ I_{V}^{*}=\frac{\lambda_{V}^{*}\Lambda_{V}\theta_{V}}{\mu_{V}\left(\lambda_{V}^{*}+\mu_{V}\right)\left(\theta_{V}+\mu_{V}\right)}. \end{array}$$

Note that

(29) $$\begin{array}{c} \lambda_{M}^{*}=\beta_{M}b_{M}\frac{I_{V}^{*}}{N_{H}^{*}},\\ \lambda_{V}^{*}=\beta_{V}b_{M}\frac{I_{M}^{*}}{N_{H}^{*}}, \end{array}$$

where *N*_*H*_^*∗*^ = *S* ^*∗*^ + *E*_*M*_^*∗*^ + *I*_*M*_^*∗*^.

Using the Center Manifold Theory [18] to study the local stability of the endemic equilibrium point (very close to *R*_0*M*_^*h*^), we have the corresponding coefficients** a** and** b** as

(30) $$\begin{array}{c} a=a_{+}-a_{-}, \end{array}$$

where

(31) $$\begin{array}{c} a_{+}=2b_{M}^{3}\beta_{M}^{2}\beta_{V}\mu^{3}\left(p+\mu +\phi \right)\left(\gamma +\mu +\delta_{M}\right)\theta_{V}\Lambda_{V}^{2}\mu_{V}{\left(\theta_{V}+\mu_{V}\right)}^{2}\left(b_{M}^{2}p^{2}\beta_{V}^{2}\mu^{2}+\left(\mu^{2}\left(p^{2}+p\left(\gamma +\mu \right)+{\left(\gamma +\mu \right)}^{2}\right)+\mu \left(p^{2}+2\mu \left(\gamma +\mu \right)+p\left(\gamma +2\mu \right)\right)\delta_{M}+\left(p^{2}+p\mu +\mu^{2}\right)\delta_{M}^{2}\right)\mu_{V}^{2}\right),\\ a_{-}=b_{M}^{3}p\beta_{M}^{2}\beta_{V}\mu^{4}\left(p+\mu +\phi \right)\left(\gamma +\mu +\delta_{M}\right)\theta_{V}\Lambda_{V}\mu_{V}^{2}{\left(\theta_{V}+\mu_{V}\right)}^{2}\left(2b_{M}p\beta_{V}\delta_{M}\Lambda_{V}+\left(p+\gamma +\mu +\delta_{M}\right)\Lambda_{H}\mu_{V}^{2}\right). \end{array}$$

Also

(32) $$\begin{array}{c} b=b_{M}p\beta_{M}\beta_{V}\mu^{2}\left(p+\mu +\phi \right)\left(\gamma +\mu +\delta_{M}\right)\theta_{V}\Lambda_{V}\left(\theta_{V}+\mu_{V}\right)\left(\delta_{M}\Lambda_{V}\mu_{V}+\Lambda_{H}\mu_{V}^{2}-2b_{M}\beta_{V}\mu \Lambda_{V}\right). \end{array}$$

The signs of the coefficients above are not known; hence we put conditions for the existence of backward bifurcation.

**Lemma 8 .**

The system exhibits backward bifurcation if the following conditions satisfy

(i) *a*_+_ > *a*_−_,
(ii) *δ*_*M*_Λ_*V*_ *μ*_*V*_ + Λ_*H*_ *μ*_*V*_^2^ > 2*b*_*M*_ *β*_*V*_ *μ*Λ_*V*_.

**Remark 9 .**

The existence of a backward bifurcation impacts the design of malaria control measures, since an epidemic may persist even *R*_0*M*_^*h*<1^.

## 5. Numerical Simulations

The full model is complicated to carry out mathematical analyses. The matrix for the full model will be 14 by 14 square matrix. Hence we only focused on the numerical simulations of the full model (1). The set of parameter values are given in Table 1.

Our model demonstrates interesting mathematical results which extend to important implications for disease management. Specifically, we show cyclical dynamics which indicate the coexistence of malaria and HIV is dependent upon the presence of both pathogens and is sensitive to a number of parameters which determine disease severity. We also demonstrate the importance of treatment for maintaining coexistence rather than disease induced extinction and further explore the role starting treatment plays in changing disease dynamic.

### 5.1. Coexistence

**Figure 3:**
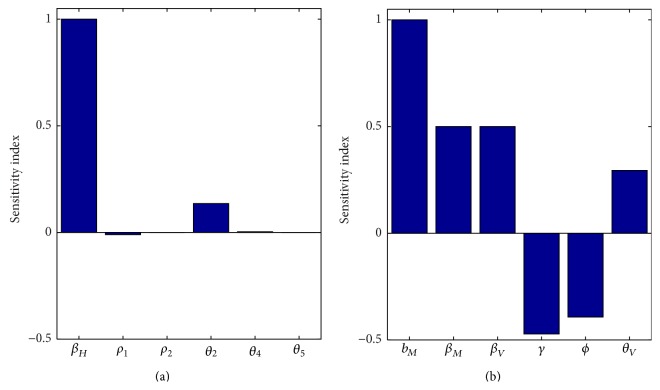
Sensitivity indices for the reproduction numbers: (a) HIV-only model and (b) malaria-only model. *R*_0*H*_ is positively affected by changes in *β*_*H*_ and *θ*_*V*_. Increasing the values of *b*_*M*_, *β*_*M*_, *β*_*V*_, and *θ*_*V*_ increases *R*_0*M*_, while changes to the parameters *γ* and *ϕ* have opposite effect on the value of *R*_0*M*_.

For our model, coexistence can only occur when the number of new malaria cases generated from a single malaria case is sufficiently low to sustain HIV infected individuals. Specifically, our simulations indicate that both HIV dynamics and malaria dynamics fluctuate but HIV dynamics synchronize with malaria dynamics such that population crashes as a result of malaria infections causing the number of HIV infected individuals to decrease. This is similar to previous work which also found the amplification effect caused by coinfection between malaria and HIV was responsible for maintaining HIV in Sub-Saharan Africa [14]. The importance of such fluctuations may further be explained when considering the sensitivities of *R*_0_ for the individual disease models with respect to their parameters. Interestingly, *R*_0_'s of our individual disease models are particularly sensitive to parameters which relate to transmission probabilities. While *R*_0_ associated with HIV is strongly and positively influenced by changes to the direct transmission parameter *β*_*H*_ (the contact rate between a human with HIV and a susceptible human), *R*_0_ associated with malaria is strongly and positively influenced by changes to the indirect transmission parameter *b*_*M*_ (the mosquito biting rate); however, *R*_0_ associated with malaria is less sensitive to the direct transmission parameters *β*_*M*_ and *β*_*V*_ (the transmission probability of malaria) but this relationship is still positive (Figure 3). Unsurprisingly, changes in parameters associated with recovery from malaria (*γ* and *ϕ*) are likely to decrease *R*_0_ associated with malaria (Figure 3(b)). Thus alterations in the individual disease transmission caused by coinfection with another pathogen are likely to increase the severity of infection of one or both diseases and alterations in the ability of an individual to recover from malaria caused by coinfection with HIV will also lead to increase in disease severity.

**Figure 4:**
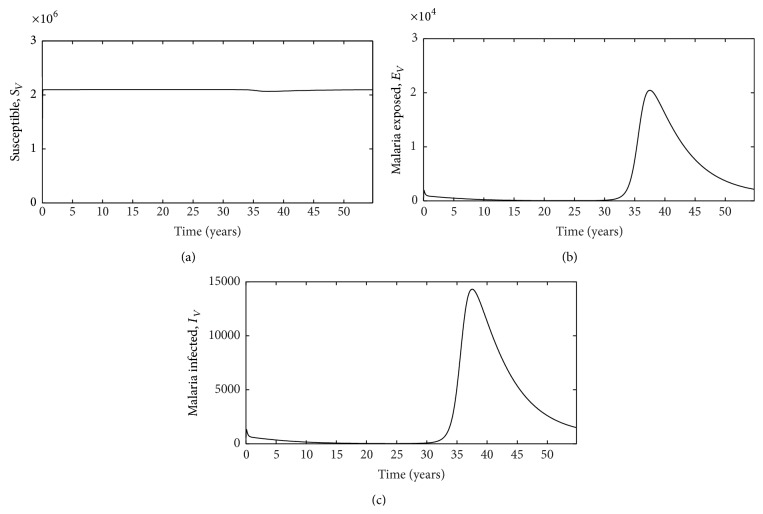
Plots for the vector population: (a) susceptible (*S*_*V*_), (b) exposed (*E*_*V*_), and (c) infected (*I*_*V*_) vectors. Here the source of malaria infection is assumed to be only from malaria infected patients.

This sensitivity to *R*_0_ may be an indicator of the role intrahost pathogen dynamics play in altering the success of multiple pathogens when individuals are coinfected. While we hypothesized that the severe impairment, HIV imposes on the host immune system, would allow malaria infection to progress more quickly [23], it appears that HIV infection actually acts to increase the rate at which individuals move from the malaria exposed class to the malaria infected class for malaria only which lowers the overall *R*_0_ for the system. This increase in disease progression may explain why malaria treatment is more likely to fail for HIV-infected individuals [24, 25] and help explain why patients infected with both malaria and HIV are more likely to experience symptoms associated with severe/complicated malaria [26], but further work is needed to confirm this mechanism. Additionally, we demonstrate similar dynamics with mosquitoes such that coinfection hinders disease progression of malaria when mosquitoes feed on hosts infected with both HIV and malaria (Figure 4). This suggests that the HIV RNA virus is interacting with the malaria parasite* Plasmodium* to alter its success in both the host and the vector, supporting previous research which indicates intrahost dynamics play an important role in explaining the effects of malaria-HIV coinfection [27].

**Figure 5:**
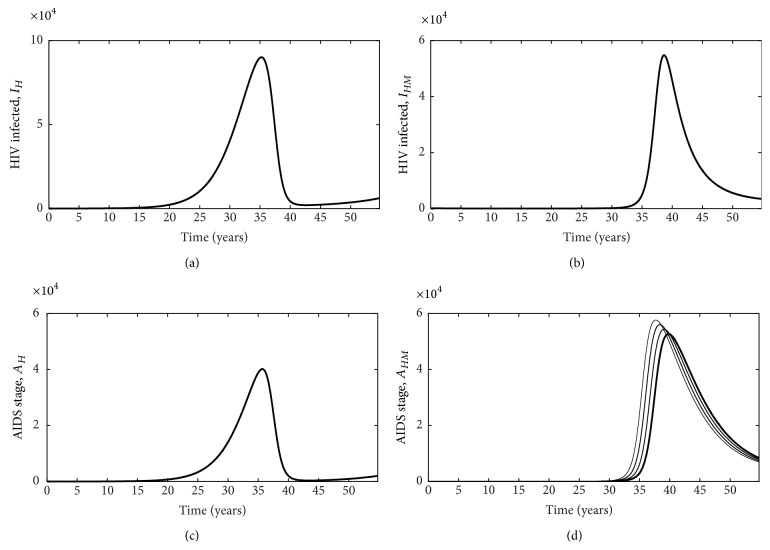
Plots for individuals not on HIV treatment as we vary the progression rate from exposed to malaria infected. HIV, *I*_*H*_ (a); coinfected with HIV and malaria, *I*_*HM*_ (b); infected with AIDS, *A*_*H*_ (c); coinfected with AIDS and malaria, *A*_*HM*_ (d). Only the number of coinfected individuals varies as we vary *ρ*. In panel (d), increases in the thickness of the line indicate increases in parameter *ρ* (progress rate from exposed to malaria infected classes).

Our model indicates an important relationship between HIV/AIDS and malaria infection such that the number of HIV infected individuals peaks earlier when individuals are singularly infected with HIV/AIDS (Figures 5(a) and 5(c)) compared to when they are coinfected with HIV or AIDS (Figures 5(b) and 5(d)). In other words, in our model, the presence of malaria delays the start of infections dynamics of HIV but speeds up the progression of the disease. This suggests that coinfection with malaria can be especially detrimental to those individuals infected with HIV/AIDS, possibly as a result of increases in viral load which result from coinfection [28].

### 5.2. Treatment

**Figure 6:**
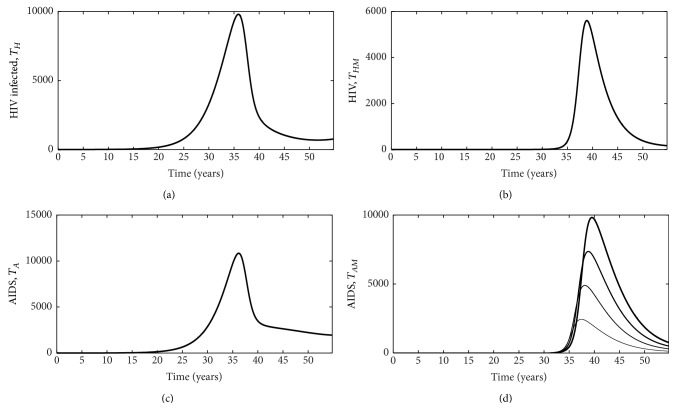
Plots for individuals receiving HIV treatment as we vary the progression rate from exposed to malaria infected. HIV, *I*_*H*_ (a); coinfected with HIV and malaria, *I*_*HM*_ (b); infected with AIDS, *A*_*H*_ (c); coinfected with AIDS and malaria, *A*_*HM*_ (d). Only the number of coinfected individuals varies as we vary *ρ*. In panel (d), increases in the thickness of the line indicate increases in parameter *ρ* (progress rate from exposed to malaria infected classes).

**Figure 7:**
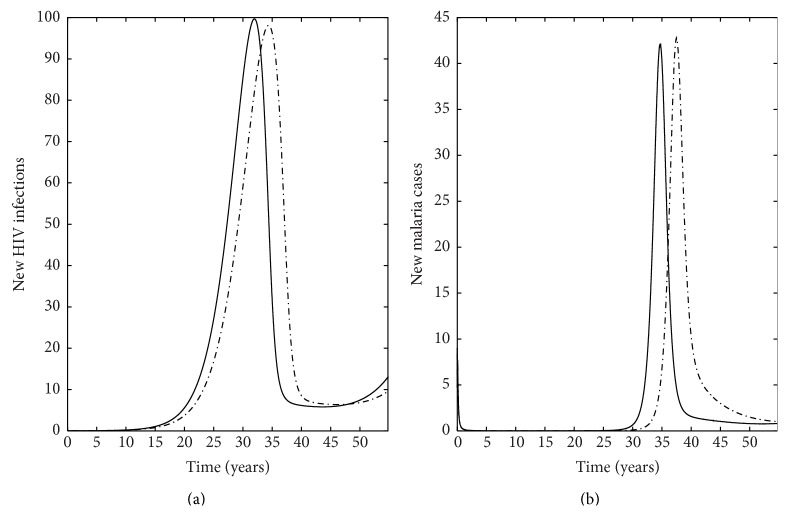
Number of new infections cases: (a) HIV infections and (b) malaria cases for individuals for two different scenarios. Solid line is a plot where there is HIV treatment, whereas the broken line is for the scenario where individuals do not receive HIV treatment. The number of new HIV infections and malaria cases is calculated by multiplying the respective force of infection with the number of susceptible individuals to HIV and malaria, respectively.

**Figure 8:**
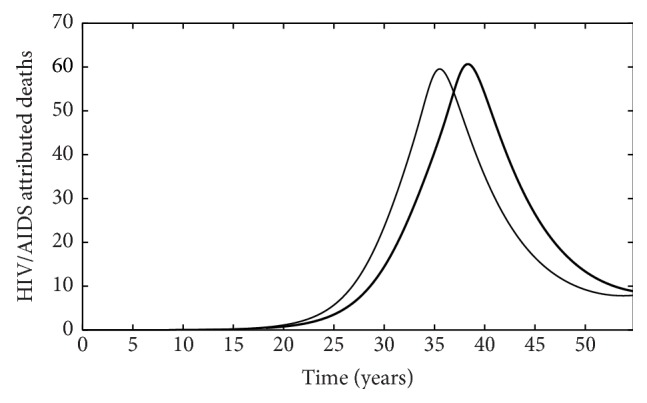
The number of HIV/AIDS attributed deaths for individuals receiving treatment (thin line) and for individuals who do not receive treatment (thick line).

Our model also has important implications for treatment as it indicates a key interaction between the rate of treatment for HIV/AIDS and malaria infection such that the number of HIV infected individuals decreases in all classes when individuals are on treatment (Figure 6) compared to when individuals are not on treatment (Figure 5). Specifically, this translates to a decrease in the number of new HIV infections (Figure 7(a)) and the number of new malaria cases (Figure 7(b)) for individuals receiving treatment compared to individuals who do not receive treatment. This is especially important for individuals coinfected with AIDS and malaria as increasing the rate individuals start treatment (*ρ*) decreases the number of infected individuals (Figure 8). Interestingly, treatment for individuals only infected with AIDS leads to a stable equilibrium earlier than when individuals with AIDS do not receive treatment (Figures 5(c) and 6(c)). Such results strongly suggest that increasing the availability and effectiveness of treatment for HIV will help dampen not only HIV/AIDS dynamics but also decrease the severity of malaria infection.

**Figure 9:**
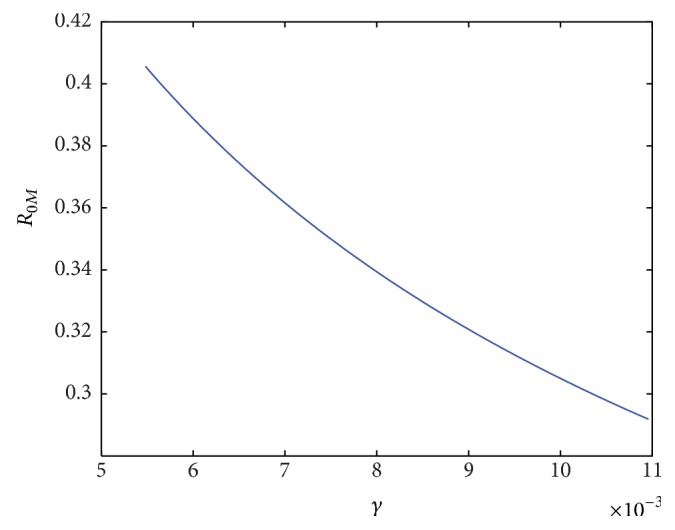
*R*_0_ specific to malaria as a function of increasing the rate at which individuals recover from malaria naturally (*γ*).

Furthermore, similar dynamics occur with malaria. While we did not specifically account for treatment of malaria, we did allow for individuals with malaria to recover naturally. Perhaps unsurprisingly, increasing this rate (*γ*) decreases *R*_0_ specific to malaria (Figure 9).

**Figure 10:**
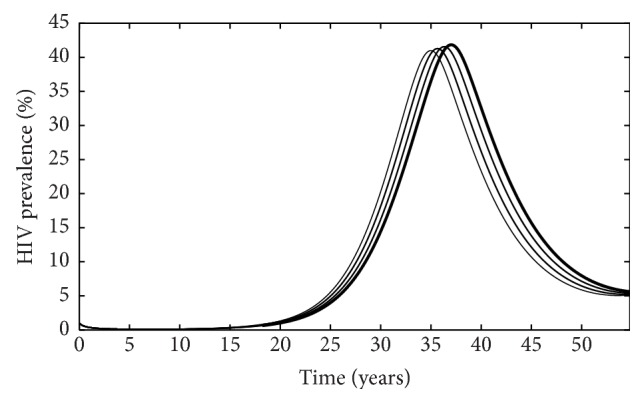
HIV prevalence (number of infected individuals/total population) as function of time. The thicker the curve, the larger the rate at which individuals start treatment (*ρ*).

When HIV is considered independently of malaria, we see that treatment does not significantly alter the HIV prevalence in the population, but it does alter disease dynamics such that increasing the rate at which individuals start treatment (*ρ*) can slightly delay increases in population prevalence over time but does not alter the duration for infection (Figure 10).

**Figure 11:**
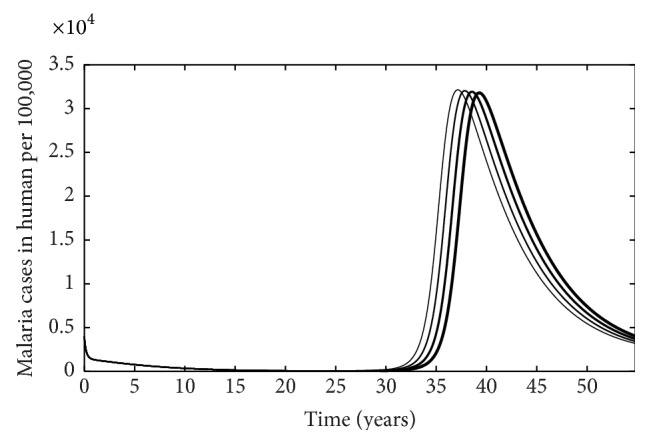
The number of malaria cases per 100,000 people as function of time. The thicker the curve, the larger the rate at which individuals start treatment (*ρ*).

Similarly, when malaria infection is considered independently of HIV, we see that treatment does not significantly alter the number of malaria cases in the population, but it does alter disease dynamics such that increasing the rate at which individuals start treatment (*ρ*) can slightly delay increases in population prevalence over time but does not alter the duration for infection (Figure 11).

## 6. Conclusion

Our model emphasizes the importance of considering HIV-malaria coinfection when considering disease dynamics in Sub-Saharan Africa. We further demonstrate amplification in HIV/AIDS prevalence as a result of malaria coinfection; however, we also demonstrate the importance of treatment for mitigating this dynamic, especially for AIDS infected patients. Therefore, we advocate for continued efforts to increase access to treatment as a means of mitigating the harmful effects of malaria-HIV coinfection.

Although our model represents a significant step forward in modeling the interaction of HIV/AIDS dynamics with malaria dynamics by including treatment, it is limited in a few ways. First, for individuals coinfected with both pathogens, HIV infection always occurs prior to acquiring malaria. This limits our ability to infer whether individuals who have malaria and then acquire HIV demonstrate the same dynamics; thus, future theoretical work is needed to explore this phenomenon. Second, our model is limited by parameterization. While we were able to acquire some of our parameter values from the literature, we had to estimate many of the other parameters. This may influence simulation dynamics. Consequently, we encourage future collaborative research with empiricists to develop experiments which will explicitly measure some of the parameters which are currently estimates in order to better inform future models.

Despite these shortcomings, the model presents some interesting results on the benefits of HIV treatment to malaria control. This is particularly important in Sub-Saharan Africa where the coexistence of Malaria and HIV/AIDS is more conspicuous.

## Figures and Tables

**Table 1:** Parameter estimation.

Parameters	Current value	Reference
Λ_*H*_	(1.932/365) *N*_*h*_0__	[19]
Λ_*V*_	6*N*_*v*_0__	[20, 21]
γ	2.5/365	Estimate
ϕ	0.2/365	Estimate
*p*	0.08333/365	[21, 22]
*w*	0.9	Estimate
μ, μ_*V*_	0.02/365, 0.1429/365	[21, 22]
δ_*M*_	3.54 × 10^−4^	[21, 22]
δ_*H*_	0.05/365	Estimate
δ_*A*_	0.2/365	Estimate
*b*_*M*_	0.15	Estimate
β_*M*_	0.5	Estimate
β_*h*_	0.567/365	Estimate
β_*V*_	0.05	Estimate
*q*	0.5/365	Estimate
σ	0.7	Estimate
α_1,2,3,4_	0.0005 each	Estimate
ρ_1,2_	0.015/365 each	Estimate
ϵ_1,2_	0.5/365, 0.05/365	Estimate
η_1,2,3,4_	1.2 each	Estimate
θ_1,2,3,4,5,6,7_	0.2 each	Estimate
*q*_1,2,3,4_	1 each	Estimate
φ, τ	0.5, 1.01	Estimate
θ_*V*_	0.1	Estimate

## Appendix

### Center Manifold Theory

We apply Center Manifold Theory (CMT) as described in [18] to establish the local stability of an endemic equilibrium point and check for the existence of backward bifurcation. It is applicable in deciding the local stability of a nonhyperbolic equilibrium (linearisation matrix which has at least one eigenvalue with a zero real part). The theory determines not only the local stability of the nonhyperbolic equilibrium but also the existence of a backward bifurcation. We duplicate Theorem  4.1 in [18] for the convenience of the reader.

Consider a general system of differential equations with parameter Φ (which will be considered as a bifurcation parameter):

(A.1) $$\begin{array}{c} \frac{dx}{dt}=f\left(x,\Phi \right),\;f:R^{n}\times R\to R^{n},\;\;f\in C^{2}\left(R^{n}\times R\right). \end{array}$$

Without loss of generality, it is assumed that 0 is an equilibrium for system for all values of parameter Φ; that is,

(A.2) $$\begin{array}{c} f\left(0,\Phi \right)\equiv 0,\;\forall \Phi . \end{array}$$

We state the theorem as follows.

Theorem A.1 A.1. Assume the following.

(1) *A* = *D*_*x*_ *f*(0,0) = ((∂*f*_*i*_/∂*x*_*j*_)(0,0)) is the linearisation matrix of system around equilibrium 0 with Φ evaluated at 0. Zero is a simple eigenvalue of A and all other eigenvalues of *A* have negative real parts.
(2) Matrix *A* has a nonnegative right eigenvector *w* and a left eigenvector *v* corresponding to the zero eigenvalue.

Let *f*_*k*_ be the *k*th component of *f* and

(A.3) $$\begin{array}{c} a=\overset{n}{\underset{k,i,j=1}{\sum }}v_{k}w_{i}w_{j}\frac{\partial^{2}f_{k}}{\partial x_{i}\partial x_{j}}\left(0,0\right),\\ b=\overset{n}{\underset{k,i=1}{\sum }}v_{k}w_{i}\frac{\partial^{2}f_{k}}{\partial x_{i}\partial \Phi }\left(0,0\right). \end{array}$$

The local dynamics of (A.1) around 0 are totally determined by a and b.

(1) Consider *a* > 0, *b* > 0. When Φ < 0 with |Φ| ≪ 1, 0 is locally asymptotically stable, and there exists a positive unstable equilibrium; when 0 < Φ ≪ 1, 0 is unstable and there exists a negative and locally asymptotically stable equilibrium.
(2) Consider *a* < 0, *b* < 0. When Φ < 0 with |Φ| ≪ 1, 0 is unstable; when 0 < Φ ≪ 1, 0 is locally asymptotically stable, and there exists a positive unstable equilibrium.
(3) Consider *a* > 0, *b* < 0. When Φ < 0 with |Φ| ≪ 1, 0 is unstable, and there exists a locally asymptotically stable negative equilibrium; when 0 < Φ ≪ 1, 0 is stable, and a positive unstable equilibrium appears.
(4) Consider *a* < 0, *b* > 0. When Φ < 0 changes from negative to positive, 0 changes its stability from stable to unstable. Correspondingly a negative unstable equilibrium becomes positive and locally asymptotically stable.

## Notations

Λ_*H*_: New recruitment to the human population
*γ*: Recovery rate from malaria infected class
*ϕ*: Recovery rate from malaria exposed class
*p*: Progress rate from exposed to malaria infected
*w*: Relative reduction to HIV infection for *I*_*M*_
*μ*, *μ*_*V*_: Natural mortality rate for human, mosquito population, respectively
*δ*_*M*_: Malaria induced mortality rate for individuals in state *I*_*M*_
*δ*_*H*_: HIV/AIDS induced mortality rate for individuals in state *I*_*H*_
*δ*_*A*_: HIV/AIDS induced mortality rate for individuals in state *A*_*H*_
*β*_*H*_: HIV transmission probability
*b*_*M*_: The biting rate of mosquitoes
*β*_*M*_: The transmission probability per bite from vector to human
*β*_*V*_: The transmission probability per bite from human to vector
*q*: Recovery rate from malaria for coinfected individuals
*σ*: A factor which accounts for differential recovery rate from malaria
*α*_1,2,3,4_: Progression rates to AIDS stage
*ρ*_1,2_: Rates at which individuals start treatment
*ϵ*_1,2_: Factors which account for differential recovery rates
*η*_1,2,3,4_: Factors which account for differential malaria infection rates
*θ*_1,2,3,4,5,6,7_: Factors indicating the differential contribution for the HIV infection rates
*q*_1,2,3,4_: Factors indicating the differential contribution for the malaria infection rates
*φ*, *τ*: Factors which account for differential AIDS induced death
*θ*_*V*_: The rate at which mosquitoes progress to infectious state.
